# Large-scale exploration of growth inhibition caused by overexpression of genomic fragments in *Saccharomyces cerevisiae*

**DOI:** 10.1186/gb-2004-5-9-r72

**Published:** 2004-08-31

**Authors:** Jeanne Boyer, Gwenaël Badis, Cécile Fairhead, Emmanuel Talla, Florence Hantraye, Emmanuelle Fabre, Gilles Fischer, Christophe Hennequin, Romain Koszul, Ingrid Lafontaine, Odile Ozier-Kalogeropoulos, Miria Ricchetti, Guy-Franck Richard, Agnès Thierry, Bernard Dujon

**Affiliations:** 1 Unité de Génétique Moléculaire des Levures (URA2171 CNRS and UFR 927 Université Pierre et Marie Curie); 2 Unité de Génétique des Interactions Macromoléculaires (URA2171 CNRS), Department of Structure and Dynamics of Genomes, Institut Pasteur, 25 rue du Dr Roux, 75724 Paris-Cedex 15, France; 3 CNRS-Laboratoire de Chimie Bactérienne, 31 Chemin Joseph Aiguier, 13402 Marseille-Cedex 20, France; 4 Laboratoire de Parasitologie, Faculté de Médecine St-Antoine, 27 rue de Chaligny, 75012 Paris, France; 5 Unité de Génétique et Biochimie du Développement, Institut Pasteur, 25 rue du Dr Roux 75724 Paris-Cedex 15, France

## Abstract

We have screened the genome of *Saccharomyces cerevisiae *for fragments that confer a growth-retardation phenotype when overexpressed in a multicopy plasmid with a tetracycline-regulatable (Tet-off) promoter. We selected 714 such fragments with a mean size of 700 base-pairs out of around 84,000 clones tested. These include 493 in-frame open reading frame fragments corresponding to 454 distinct genes (of which 91 are of unknown function), and 162 out-of-frame, antisense and intergenic genomic fragments, representing the largest collection of toxic inserts published so far in yeast.

## Abstract 2

A screen of the *Saccharomyces cerevisiae* genome for fragments conferring a growth-impairment phenotype identified 714 fragments in about 84,000 clones tested.

## Background

The complete genome sequences of various eukaryotic model organisms such as *Saccharomyces cerevisiae*, *Caenorhabditis elegans*, *Drosophila melanogaster*, *Arabidopsis thaliana *and *Schizosaccharomyces pombe*, have revealed a large number of novel genes of unknown functions. In *S. cerevisiae*, for example, around 1,800 genes (of the total of around 5,800) encode proteins that so far remain functionally uncharacterized (compilation from *Saccharomyces *Genome Database (SGD) [1] April 2004). Since the completion of its DNA sequence [2], the genome of *S. cerevisiae *has been extensively studied, serving as a test case for novel and important developments in functional genomics. Such developments include transposon-mediated gene inactivation and tagging [3], the analysis of gene-expression networks through partial or complete transcriptome studies [4-6], two-hybrid screening [7-9], protein-complex purification [10,11], two-dimensional gel protein identification [12], proteome qualitative analysis by protein microarrays (see review in [13]) and protein abundance measurements after *in situ *gene tagging [14]. Even intergenic regions have been studied using microarray technology to characterize transcription-factor-binding sites and to map replication origins or recombination hotspots [15,16] (see also [17] for a review). Following a large cooperative effort between European and American labs, a nearly complete collection of deletion mutants of all yeast protein-coding genes is now available [18-20], which offers the possibility of systematically screening numerous phenotypes, including synthetic lethals [21-23], in search of novel gene functions.

As a complement to gene inactivation, phenotypic changes resulting from gene overexpression may also be informative of gene functions. Indeed, in a number of cases, such as genes encoding cytoskeletal proteins or protein kinases and phosphatases, overexpression may lead to a lethal phenotype (see [24] for a review). The overexpression approach is complementary to the loss-of-function approach, as it leads to dominant phenotypes even in the presence of the wild-type gene, thus allowing the study of genes for which no loss-of-function mutants can be obtained. Overexpression of gene fragments can be equivalent to 'dominant negative mutation' in which the fragment disrupts the activity of the wild-type gene [25]. Overexpression can also activate specific pathways, leading to deleterious phenotypes: examples include genes involved in the yeast pheromone response pathway, such as *STE4*, *STE11 *and *STE12 *(see [24,26] and references therein). In other cases, specific effects are not known, but the region responsible for toxicity has been identified. For example, lethality upon overexpression of Rap1p depends on the presence of the DNA-binding domain and an adjacent region [27]. In general, however, unless the domain structure of the protein is well understood, one cannot predict which segment(s) of it would act as a dominant mutant when overexpressed.

Several yeast cDNA libraries have been screened for lethal or impaired growth phenotypes upon overexpression under the control of the *GAL1 *or *GAL10 *promoters on centromeric or multicopy plasmids [28-30]. Other libraries of random genomic DNA have also been screened for toxicity upon overexpression from the same promoters [24,26]. Whereas the four earlier studies each identified only a few genes (from 1 to 24 each, making a grand total of 43), Stevenson *et al. *[30] identified 185 genes (20 of which were shared with earlier work) that cause impaired growth when overexpressed.

In the work reported here, we have screened the yeast genome with the aim of characterizing a list of fragments whose overexpression confers growth impairment. To do this, we constructed a yeast genomic library in a multicopy plasmid vector in which transcription is driven by a chimeric tetO-*CYC1 *promoter [31]. Random genomic inserts of a mean size of 700 base-pairs (bp) were overexpressed in yeast as translational fusions using the plasmid-borne initiation codon. Out of around 84,000 clones tested, we have identified the largest collection yet of toxic overexpressed fragments in yeast: 714 showed overexpression-dependent lethality or various degrees of growth impairments, identifying 454 protein-coding genes (91 of which are of unknown functions), and a variety of intergenic or other regions.

## Results

### Screening the library of yeast random genomic fragments for toxic phenotypes

**Figure 1:**
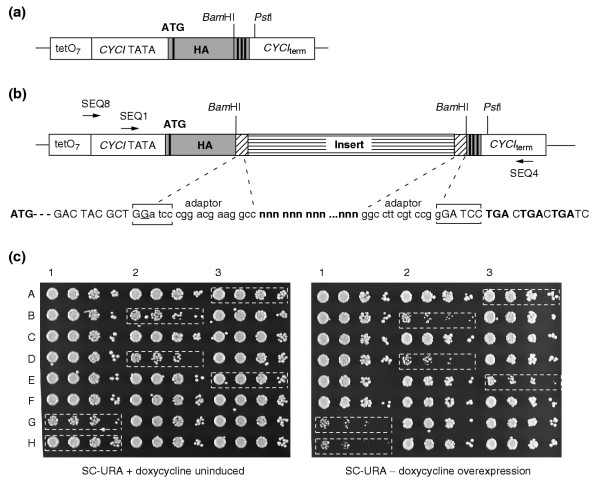
Overexpression library construction and screening. **(a) **Construction of an HA-tagged vector. The pCMha190 vector used here was constructed by insertion of a linker (gray box) in place of the multiple cloning site in vector pCM190 [31]. Features shown include the promoter and TATA box as well as the terminator from the original plasmid (open boxes), and the start codon, HA-tag, *Bam*HI site and stop codons (thick vertical bars) from the introduced linker sequence. The linker was composed from the following annealed oligonucleotides: EXP3: 5'-GATCGTTTAAACCATATGTACCCATACGACGTCCCAGACTACGCTGG ATCCTGACTGACTGATC-3', EXP4: 5'-GGCCGATCAGTCAGTCAGGATCCAGCGT AGTCTGGGACGTCGTATGGGTACATATGGTTTAAAC-3'. **(b) **Library construction in pCMha190 (see Materials and methods for experimental details). The resulting ligation product is schematized, with the insert as a striped box and adaptors as hatched boxes. Sequences shown below are from junctions, with uppercase letters corresponding to vector (the extra nucleotide from filling-in is underlined), lowercase letters to adaptors and bold nnn's to insert. Arrows indicate the different primers used: SEQ8 and SEQ4 are used for PCR amplification of the insert, and SEQ1 for sequencing (see sequences in Additional data file 8). **(c) **First-round screening of toxic phenotypes. The growth of random and control clones on selective medium in uninduced and overexpression conditions is shown. Drops of serial dilutions (1/100 to 1/100,000) of cultures were grown for 45 h at 30°C. A3, non-toxic control clone transformed by pCMha190; H1, toxic control clone transformed by *MCM1 *gene cloned in pCMha190; G1, B2, D2, E3, library transformed clones, exhibiting different levels of toxicity in overexpression conditions (see Figure 2).

**Figure 2:**
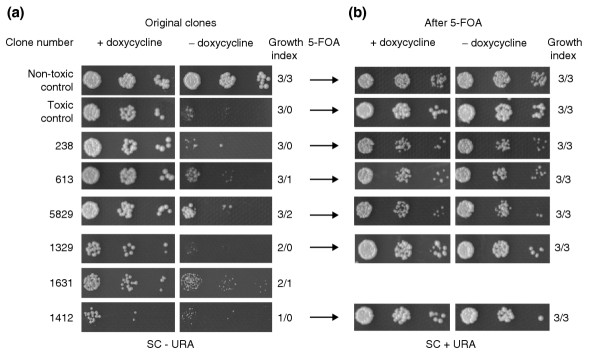
Second-round scoring of toxic phenotypes and control. **(a) **Selected clones from the first round were diluted and three drops (1/100, 1/1,000 and 1/10,000) were spotted and grown for 42 h at 30°C, with controls on same plates, for confirmation of toxicity. Growth levels in the presence and absence of doxycycline were scored as described in the text. Each clone was assigned a growth index where the first number represents the growth in uninduced conditions and second number the growth in induced conditions; for example, 3/3 indicates a non-toxic insert; 3/0 indicates a highly toxic insert. Clone numbers are the same as in the tables describing the toxic inserts (see Additional file 1,2,3,4). **(b) **After 5-FOA-induced plasmid loss, growth of surviving clones is scored in the same way as in (a). Wild-type phenotypes in overexpression conditions are indicative of plasmid-borne toxicity.

We have analyzed a total of 84,086 independent yeast transformants, each of which contains a random fragment of the yeast genome placed under the control of a doxycyclin-repressible promoter (Figure 1a,1b). Effects on growth or survival were monitored by spotting serial dilutions of the transformants in the presence and absence of doxycyclin (uninduced and overexpression conditions respectively, Figure 1c). Phenotypes were recorded using numerical values from 0 to 3 (Figure 2): value 3 was assigned to normal growth (similar to non-toxic control), 2 and 1 were assigned to intermediate growth levels (less abundant and/or smaller-sized colonies), and 0 was assigned to complete or almost complete absence of colonies (comparable to the toxic control on the same plate). We have retained 714 clones (0.85% of total) that show impaired growth in overexpression conditions (Table 1). Among these, 112 also show a slight or severe growth reduction (level 2 for 77 cases, or level 1 for 35 cases, respectively) in unexpressed conditions. Proof that the observed growth defects were caused by the presence of the plasmid rather than an accidental mutation in the clone was directly demonstrated by the recovery of the wild-type phenotype after plasmid loss using selection for resistance to 5-fluoroorotic acid (5-FOA) (Figure 2).

### Identification of the genomic inserts conferring toxic phenotypes

Inserts of the selected clones were identified by DNA sequencing (Materials and methods). The complete list of inserts is described in Additional file 1 and 2, and results are summarized in Table 1. A majority of inserts (493, or 69% of total) carry in-frame portions of annotated open reading frames (ORFs), excluding Ty and Y' ORFs. In addition, a significant number of inserts (162 (23%)) correspond to fragments of ORFs cloned either in antiparallel orientation or out-of-frame with respect to the initiator ATG codon or to intergenic regions. The 59 remaining cases (8% of total) correspond to fragments of transposable elements (17 clones) and subtelomeric Y' elements (9 clones), to RNA-coding genes (4 clones), and to non-chromosomal replicons such as the 2 mm plasmid and mitochondrial DNA (mtDNA) (29 clones). If any random fragment of the yeast genome were capable of generating a toxic phenotype, in-frame ORF fusions would represent only around 10-12% of the selected inserts (around 70% of the genome correspond to coding regions, and only one frame out of six corresponds to the natural frame). The fact that the toxic inserts correspond principally to in-frame portions of natural ORFs suggests that the coding part of the genome is the most prone to confer toxicity when overexpressed.

### Analysis of domains within in-frame ORF fragments

The 493 inserts corresponding to in-frame ORF fragments represent 454 distinct annotated ORFs (see Materials and methods), which are randomly distributed throughout the 16 chromosomes of *S. cerevisiae *(see Additional file 1). In our screening, 32 ORFs were found twice, two ORFs were found three times and one ORF (*YHR056*c in the *CUP1 *region) was found four times, the cloned fragments being either overlapping (22 ORFs) or non-overlapping (13 ORFs). Mean size of the coding region of inserts is 659 bp. The chosen cloning strategy favors recovery of central-or carboxy-terminal coding parts of the natural yeast genes, whereas the amino-terminal coding regions are rare [7]. In our work, the cloned insert encompasses the entire gene in only six cases (additional file 3, column 20 to 23). In 154 additional cases, the insert corresponds to the carboxy-terminal portion of the natural protein (the stop codon is present). In 10 cases, the inserts start upstream of the natural ATG initiator codons, lengthening the natural peptides by reading in-frame through the untranslated region. Other cases correspond to the central coding region of natural genes.

To find possible common characteristics, we have compared between themselves all the peptides encoded by in-frame ORF fragments. BLASTP analysis was combined with detection of characterized conserved domains, of COG patterns (clusters of predicted orthologous groups of proteins [32]), and of transmembrane spans (TMS) to identify toxic inserts similar to each other (see Materials and methods). Out of the 493 in-frame ORF fragments, a total of 170 were divided up into 57 distinct groups of similarity, containing from two to 12 inserts, including overlapping fragments of the same ORF (see Additional file 4). It is expected that several ORFs from a same paralogous gene family are found in a same group. Note that in 16 out of 57 groups, the inserts contain transport-specific domains and/or transmembrane spans.

As well as comparing inserts to each other, we also analyzed the totality of the conserved domains present in all peptides encoded by the 493 toxic inserts (see Materials and methods). Characterized domains are found, at least partially, in a total of 281 inserts (see additional file 1 and 3). Of a total of 183 distinct domains, 46 are represented more than once. We have compared the frequency of these 46 domains among the toxic inserts versus their frequency among the 5,803 ORF-encoded proteins of the entire genome (Table 2). We find that 37 domains are significantly over-represented compared to a random expectation, suggesting that we have screened specific domains.

These 37 domains correspond predominantly to various transporter domains (11 cases), such as amino-acid permeases and mitochondrial carrier protein domains. The toxicity of these domains is probably due to the presence of transmembrane spans. Indeed, 132 out of the 493 toxic peptides contain at least two transmembrane spans, including cases where one span is putative (see Materials and methods). Among these, 63 contain three or more predicted spans and 26 have five spans or more. Putative spans were also recognized in 84 other ORF fragments (seven with at least three spans, 15 with two spans, and 62 with one span) (see Additional file 1 and 3).

RNA-and DNA-binding domains (nine cases) involved in replication, transcription or translation functions, such as PUF, KH and rrm, are also much more represented than expected (Table 2). The PUF domain is also involved in recruitment of proteins into a complex that controls mRNA translation (see [33] for review).

Other important domains for interactions with polypeptides, phospholipids or small molecules (nine cases) are also over-represented. The WD40 motif, a propeller-like platform for stable or reversible binding of proteins in eukaryotes, has been found in inserts of 12 distinct ORFs (see additional data file 1). The 12 ORFs code for proteins having interactions with other proteins in complexes related to RNA processing or transcription [10], and nine have at least one partner also selected during our screening (see Discussion). Other interacting domains were found, such as dynamin, MRS6, and adaptin_N domains, which have roles in the dynamics of proteins, membranes and cytoskeleton, and PBD, a small domain which binds small GTPases and inhibits transcription activation. The PH domain, which binds phosphoinositides or other ligands and is involved in signal transduction, was found in inserts of three distinct ORFs involved in different functions: metabolism, cell fate, transcription (see Additional data file 3). Finally, other over-represented domains are related to metabolism and other functions (eight cases), of which several may be involved in interactions with other domains.

The serine/threonine protein kinase domain (S_TKc) is significantly under-represented in our screen. Among the 10 toxic inserts whose cognate genes code for protein kinases (PK), only four contain this domain (Additional data file 3). In these four cases, the S_TKc domain is either truncated (Additional data file 4), or flanked by a coiled-coil region and/or a low-complexity segment. Two other inserts contain the PBD (and PH) domains, and the four remaining inserts contain no characterized domain to date. As it is known that overexpression of some protein kinases is deleterious for cells (see [24] and references therein), our results suggest that a domain different from the catalytic domain is responsible for the toxicity of these proteins, and that the fragments selected in our screen have a role in binding ligands such as substrates or regulators of protein kinase activity, or of proteins involved in the signaling cascades. Three other genes coding for protein kinases of the phosphatidylinositol 3-kinase (PI kinase) family are also represented in our screen by four toxic inserts, none of which contained the kinase domain (see Discussion).

The remaining 137 domains (out of 183) were found only once each. Many correspond to functional categories described above, such as transport, metabolism, and interactions with nucleotides, other proteins or other ligands. Seven domains associated with ubiquitination functions were also found (see Additional data file 3 and 5). Several of the domains encountered have also been isolated as mammalian genetic suppressor elements (GSEs), which are cDNA fragments that inhibit cell growth (see [34] and references therein).

In addition to the domains described above, we found toxic inserts coding for natural peptides without recognizable domains but containing regions of low complexity (56 cases). A number of these peptides are highly charged, either negatively or positively (see Additional data file 3). Such charged peptides might interact in an artifactual way with other charged domains of proteins or nucleic acids or with small molecules. Interestingly, the prion-like (Q+N)-rich domain was found in eight of the natural peptides having low-complexity regions.

### Nature of the selected genes

We have seen above that 493/714 toxic inserts are in-frame fragments of protein-coding genes. The complete list of the 454 genes corresponding to these toxic inserts is given in Additional dat file 1 and 2. Their sizes range between 282 bp and 14,733 bp. The mean size of this distribution is 2,401 bp (standard deviation (SD) 1,671 bp), to be compared with a mean size of 1,444 bp (SD 1,094 bp) for the entire set of 5,803 ORFs of the yeast genome. The bias towards longer ORFs is expected from our cloning strategy (see above). Note that the 35 ORFs that we found more than once are nearly randomly distributed in various size classes.

We examined the distribution of these genes according to different criteria, such as function, subcellular localization, viability and phylogeny (Table 3) and compared it to the distribution of the genes of *S. cerevisiae*.

Among the 454 ORFs identified, 91 are unclassified, and function is not yet clear for six others (see Additional data file 3). The remaining ORFs represent a variety of functional classes (Table 3). Distribution of the 454 ORFs shows statistically significant deviations for eight out of the 15 functional classes, taking into account biases due to mean size of genes in each class. Globally, there is a deficit of genes involved in protein synthesis and of unclassified genes, and an excess of genes involved in transport facilitation and cellular transport (echoing the fact that we found many inserts containing transporter domains and transmembrane spans), in cell fate, in transcription and, to a lesser extent, in cell cycle/DNA processing and in homeostasis (regulation of/interaction with the environment).

As seen above, many toxic inserts contain multiple predicted TMS. Such inserts correspond most often to genes coding for transporters or for non-transporter membrane proteins [35]. We have selected a total of 96 transporters (see Additional data file 3) of which 18 belong to the class of putative uncharacterized transporters, whose toxic inserts contain several TMS. Fourteen others belong to the class of transporters of unknown classification, including 13 genes of the nuclear-pore complex family, whereas there is a total of 58 genes in this family in the whole genome. On the other hand, 24 genes coding for non-transporter membrane proteins were also selected. Taken together, 120 transporters and non-transporter membrane proteins are represented in our screen, twice as many as expected (61 expected), as 782/5,803 ORFs are known or predicted as coding for such proteins [35].

The distribution of the proteins encoded by these genes in the cell is strongly biased in favour of the plasma membrane and against the cytoplasm, and, to a lesser extent, in favour of nucleus and cytoskeleton (Table 3).

Although the majority of inserts originate from non-essential genes, we have found 96 essential genes (21%) among the selected ORFs. This is a significantly higher percentage than in the whole genome, where 939/5,803 genes (16.2%) are essential (Table 3).

Using the classification from Malpertuy *et al. *[36] and additional updating (Génolevures [37]), we find that the majority of genes yielding toxic fragments in this work are conserved (336/454 (74%)) between *S. cerevisiae *and other sequenced organisms, whereas 106 (23%) are ascomycete-specific and 10 (2.2%) are orphan genes. This distribution is significantly different from the distribution among the 5,803 genes of *S. cerevisiae*, where 64% of protein-coding genes are conserved (see Table 3). The under-representation of orphan genes in our screen is already apparent in the under-representation of functionally unclassified genes, as a high rate of orphans of the whole genome (79%) are also unclassified (data from Génolevures [37] and Munich Information Center for Protein Sequences (MIPS) [38]).

### Toxicity of entire genes versus ORF fragments

**Figure 3:**
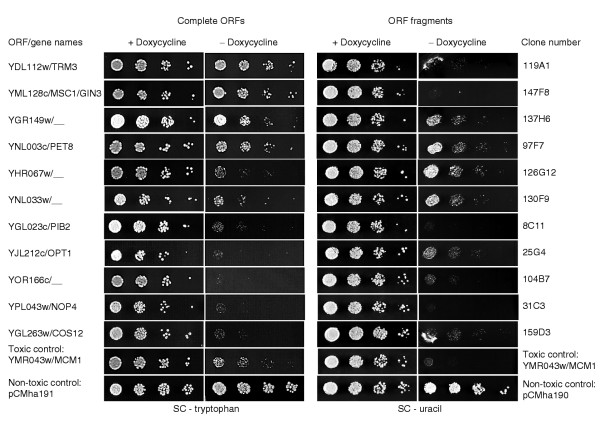
Toxic phenotypes of overexpressed fragments versus whole ORF products. Complete ORFs are cloned in pCMha191 (tryptophan marker); inserts are cloned in pCMha190 (uracil marker). Eleven out of the 13 cases are represented in this figure. + doxycycline, uninduced conditions; - doxycycline, overexpressed conditions.

To compare the phenotypes conferred by overexpression of the entire gene and of the gene fragment, we have cloned the cognate entire genes of 13 in-frame toxic inserts into the vector pCMha191 (see Materials and methods). One criterion for the choice of the genes was the absence of a mutant phenotype of the corresponding gene disruption at the time this work was started, except for the *NOP4 *gene whose disruption is lethal. Six of these genes are singletons; three others have a paralog already known as toxic upon overexpression. Six out of the 13 still have no known function to date (Table 4). Expression at the protein level of both entire gene and gene fragment was verified by western-blot analysis, using an anti-hemagglutinin (HA) antibody (data not shown). As seen in Table 4 and Figure 3, we found that overexpression of 10 genes was as toxic or more toxic than overexpression of the gene fragments. One gene, *YGR149w*, was less toxic in its entire version than in the truncated form, which was weakly toxic. Finally, we found that two genes, *YML128c*/*MSC1 *and *YDL112w/TRM3*, showed no toxicity when overexpressed, whereas the cloned inserts were strongly toxic. In these two cases, the immunolocalization of overexpressed products was examined, and the cytoplasmic localization of the fragment agreed with the location of the natural gene product (data not shown), indicating that the toxic effect is not the result of mislocalization of the overexpressed fragment. The gene *MSC1 *had already been screened [24] as a toxic fragment in overexpression conditions, the region concerned being the same as in our screening. This gene has low similarity to a stress protein of *Schizosaccharomyces pombe *and has a role in meiotic recombination. The *TRM3 *gene contains a carboxy-terminal domain responsible for tRNA methyltransferase activity [39], which is absent from our insert. The protein is a member of a complex probably involved in signaling [10].

### Analysis of other fragments

Additional data file 2 analyzes the 221 other toxic inserts which do not correspond to in-frame fragments of annotated ORFs. Sixty-eight inserts correspond to natural ORF fragments cloned in an antiparallel orientation, most of them being entirely included within the ORF sequence (47 cases), the others overlapping the intergenic upstream region of the natural ORF (17 cases) and sometimes the next gene as well (four cases). Their toxicity can result either from the overexpression of an antisense RNA or from the overexpression of a toxic artificial peptide encoded by a fortuitous ORF. Several arguments favor the second hypothesis. First, short ORFs longer than 24 codons (maximum observed 250 codons), and in-frame with the start codon of the cloning vector, are observed in 53 cases (78% of the total). A number of those artificial ORFs are due to the 'mirror' effect produced by codon-biased natural ORFs [40,41]. But the fact that they are observed more than one-third of the time suggests a positive selection for toxic artificial peptides. Second, antiparallel ORF fragments do not correspond to a majority of essential genes, as might be expected from antisense RNA inhibition. Third, we have directly verified, for two inserts recloned in the same vector, that addition of a stop codon that blocks translation of the artificial ORF also suppresses toxicity (see Additional data file 9). Even if this concerns only two cases, we have no direct results indicating the existence of antisense RNA molecules that could block expression of essential genes.

Fifty-three additional inserts correspond to natural ORF fragments cloned out-of-frame with respect to the plasmid-borne ATG codon, of which only 12 code for artificial ORFs longer than 24 codons (see Table 1 and Additional data file 2). Intergenic regions are represented by 41 inserts, of which 27 (65% of total) code for short artificial ORFs.

In total, short artificial peptides may be encoded by 92 out of the 162 inserts described above. Comparison of the 92 peptides between themselves reveals several low-complexity sequences (see Additional data file 2), mostly encoded by antiparallel ORF fragments whose direct amino-acid sequence is itself of low complexity. Comparison with the proteins of *S. cerevisiae *and of all available sequenced organisms compiled in our internal database (GPROTEOME3, see Materials and methods) reveals no significant similarity. None of these artificial ORFs corresponds to the 137 new annotated yeast genes of Kumar *et al. *[42], to the 62 new genes of Oshiro *et al. *[43] or to the 84 genes of Kessler *et al. *[44]. Even though we have no evidence for antisense RNA activity, we cannot exclude a toxic effect due to the overexpressed transcript itself.

Among the 59 remaining inserts, 17 belong to Ty elements, 10 of which are in-frame ORF fragments corresponding to TyB only (two of them containing the carboxy-terminal part of the rve domain (integrase core)), whereas all antisense fragments (three inserts) correspond to TyA. Y' elements, which are present in 20 copies in the genome, are represented by nine inserts, all coding for highly basic or acidic peptides (of which three are in-frame fragments of natural ORFs) which contain repeats of amino acids or motifs, and confer a strongly toxic effect (see Additional data file 2). Considering that these inserts are toxic, their observed number is not different from that expected from the size and number of Y' in the genome.

Four inserts from yeast chromosome XII are fragments of genes coding for 18S or 25S RNA, two inserts being cloned in the sense orientation. The 2 mm plasmid is represented by 17 fragments, 10 of which are in-frame fragments of ORFs coding for *REP1*, *REP2 *and *FLP1*. The seven other inserts are out-of-frame or antisense fragments of *FLP1*, or fragments of intergenic regions, all (except two) coding for artificial ORFs. Finally, mtDNA is represented by 12 fragments, mostly corresponding to intergenic regions on the minus strand of the chromosome. Artificial peptides highly enriched in the amino acids tyrosine (Y), isoleucine (I), and lysine (K) are encoded by 10 out of the 12 mitochondrial inserts.

## Discussion

The general fitness of living organisms largely depends on a harmonious equilibrium between the various cellular components and on their capacity to maintain homeostasis. The intricate circuitries that regulate gene expression form the basis of these properties, and massive deregulation of single components may result in flagrant phenotypic defects leading to serious growth impairment or even cell death. Our large-scale screening of the yeast genome using random genomic fragments resulted in a collection of several hundreds of inserts showing toxic effects on cell survival or growth when overexpressed. These toxic effects are expected to result from several distinct molecular situations that have been encountered at various frequencies in our experiments. Of the total of 714 toxic inserts studied, a majority (69%) correspond to the overexpression of fragments originating from natural protein-coding genes (454 genes were identified in total). But, interestingly, a large minority (23%) correspond to noncoding DNA fragments. The remaining cases (less than 10% of the total) correspond to fragments of Ty or Y' elements, of the 2 μm plasmid or of mtDNA which, after analysis, can be attributed to one of the two previous categories. Toxic fragments of natural gene products are interesting to consider with respect to the functions of the corresponding genes. But the second category may be even more promising in that it offers us a description of DNA sequences that cannot be overexpressed in a cell without a deleterious effect.

**Figure 4:**
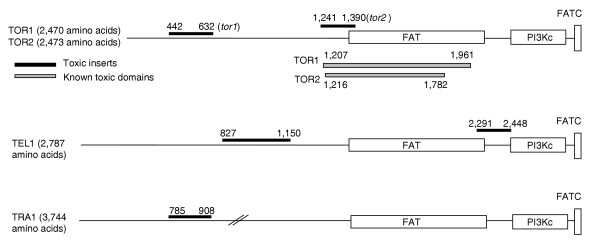
Positions of selected toxic fragments relative to the structure of genes of the PI kinase family. Names of the selected genes and protein lengths (in amino acids) are indicated. Coordinates of the toxic fragments selected in this work and of known toxic domains (see text) are also given. Conserved domains in the proteins have been positioned using the NCBI CD-Search program [64] (see Materials and methods). Domain abbreviations: FAT (pfam 00259) is named after FRAP, ATM and TRRAP, which are human homologs of yeast TOR, TEL1 and TRA1, respectively; PI3Kc (smart00146) is the PI kinase catalytic domain; FATC (pfam02260.11) is named after FRAP, ATM, TRRAP carboxy-terminal region. Complete COG5032 TEL1 (2,105 residues) spans the carboxy-terminal regions of the four proteins. The drawing is not to scale.

The toxicity of coding fragments may result from the imbalance between products of tightly controlled genes, or from the titration of active complexes by the presence of truncated proteins and/or isolated domains. In addition, nonspecific effects might also exist, for example, as a result of an abnormal intracellular localization of an artificially overabundant peptide or protein. We did not attempt to distinguish experimentally between these possibilities for all the coding inserts isolated in this work. Taking into account only specific effects, in the limited number of cases in which the entire gene corresponding to a toxic insert was cloned in the overexpression vector (see Results), we verified that toxicity was due, in most cases, to the disruption of the precise dosage of an essential cellular component (the entire protein is also toxic when overexpressed) and, in some cases, to the titration effect exerted by the incomplete fragment of the natural protein (the entire protein is not toxic when overexpressed). A few examples where the domain responsible for toxicity upon overexpression is known can be found in the literature. In the case of *TOR1 *and *TOR2 *genes, toxicity is specific to a central domain of the proteins distinct from their carboxy-terminal protein kinase domain; overexpression of the entire gene has no effect, and can even cure the negative effect of the overexpressed domain [45]. Alarcon *et al. *[45] have proposed that Tor proteins could serve as a scaffold on which to assemble other proteins for appropriate interaction with the kinase domain. Our results agree with this hypothesis, as four out of the five yeast genes belonging to the conserved family of PI kinase-related protein kinases - *TOR1*, *TOR2*, *TEL1 *and *TRA1 *- were selected in this work, all represented by inserts of the central region of these proteins (Figure 4 and Additional data file 3). In mammalian cells, overexpression of such fragments of *ATM*, a homolog of *TEL1*, also has a negative effect [46]. In other cases in which overexpression of the entire gene is toxic, certain domains responsible for the toxicity have been mapped, for example the Myb DNA-binding domain of *RAP1 *(see Background), the ZnF C3H1 domain of *CTH1 *[47] and the bZIP domain of *GCN4 *[48]. All these DNA-binding domains were significantly over-represented in our screen (Table 2).

Even in the absence of precise mapping of the toxic domain present in our clones, we were able to explore the nature of the domains found in each insert. Our experiment has shown a bias towards domains corresponding to transport functions and to various interactions (Table 2). As mentioned in Results, the toxic effect of transport-specific domains may be due to the presence of corresponding TMS.

As our results also showed a bias towards a number of interaction domains, we have examined the known interactions of the proteins encoded by the 454 genes found in this screen (see Materials and methods). Genetic interactions were also considered, excluding the coexpression results obtained in microarray experiments. It appears that 88.3% of our genes (401/454, of which 70 are of unknown function) code for proteins which have known genetic or physical interactions, or are members of complexes (see Additional data file 3). Moreover, for 60% of these (242/401), at least one of their known partners is also found in our screen (see Additional data file 6 and 7). Among the 53 genes having no known interactions, 24 correspond to transporter or membrane proteins (see Additional data file 3).

The biases we have observed show little overlap with previous screenings of *S. cerevisiae*, which had previously identified a total 231 genes or gene fragments that were toxic when overexpressed [24,26,28-30,49]. Among the 185 genes of Stevenson *et al. *[30], those involved in protein synthesis are represented twice as frequently as in the whole genome, whereas they are twice less frequent in our own experiment. In contrast, genes involved in transport facilitation and interactions with the environment were not over-represented in the Stevenson *et al. *experiment. Common biases are, however, observed in favor of transcription, cell-cycle and cellular transport genes. Overall, only 33 of our 454 ORFs were previously identified by the previous authors (the total rises to 78 if one considers individual gene studies). Twenty-five other genes from the previous screenings not found here are members of paralogous gene families represented in our work (see Additional data file 3). The limited overlap may result from partial genome coverage. However, by screening 84,086 clones (a coverage of around 4.5 genome equivalents), we must have encountered a total of 4,677 ORFs, each being represented 1.6 times as an ORF fragment (see Materials and methods). We have thus screened for toxicity around 80% of the natural yeast ORFs. But the limited overlap of results may also be explained by the experimental bias introduced by each technique. The previous experiments were mostly based on cDNA cloning, which favors short and highly expressed genes, whereas our genomic library favors large ORFs (mean size 800 ± 557 codons per ORF) and has no expression bias. In addition, the largest previous experiment [30] was done using centromeric plasmids and a galactose promoter as opposed to our multicopy vectors. Furthermore, our serial dilution drop assay is probably more sensitive to growth alteration than the replica techniques previously used. Finally, previous overexpression experiments relied on changing the nutrient composition of the growth medium (galactose vs glucose) whereas our experimental set-up relied on the presence/absence of a drug in a medium of the same nutritional composition.

The finding of a large minority of toxic inserts corresponding to noncoding DNA is puzzling. Indeed, some of the toxic inserts originate from annotated but questionable ORFs, and some originate from antisense or intergenic fragments which can artificially be translated into small ORFs. None of these peptides has recognizable characterized domains, but many of them are charged, mostly positively (see Additional data file 2) and some have amino-acid sequences of low complexity. It could be proposed that all these small ORFs represent a reservoir of potentially new gene sequences in the genome. In addition, 100 of the in-frame toxic inserts had no characterized domains and sometimes no predicted secondary structure. These inserts do not contain conserved domains, COGs or TMS, and are not biased in amino-acid composition (see Additional data file 3). They may correspond to domains that have not yet been described, or to domains whose structure has diverged, but another possibility would be that some protein domains are perhaps not structured in a permanent way before evolving towards a structurally functional domain. Interestingly, a significant proportion of the expressed peptides we selected are specific to ascomycetes, or are even true orphan genes that have no known homolog in any other species than *S. cerevisiae*. A collection of toxic polypeptides, acting as genetic suppressor elements and interfering with major cellular functions, is of interest not only in antifungal research but also as a means of identifying new domains with major physiological roles.

Finally, given the large number of inserts encoding very short ORFs (around 70 amino acids in the groups of antiparallel, intergene and out-of-frame fragments, and 80 in total, see Additional data file 2), we cannot exclude the possibility that some transcripts are toxic through hypothetical mechanisms that may include, for example, nonspecific interactions with other cellular or nuclear complexes or through overloading of some component(s) involved in RNA metabolism.

## Conclusions

In a large-scale phenotypic screening of overexpressed random DNA fragments, we selected around 470 genes (including Ty, Y' and the 2 μm plasmid) whose domains inhibit or impair growth when overexpressed. Many functional categories are represented, transporter proteins being especially over-represented, and genes of unknown function represent one-fifth of our selection. Our approach gave access to genes controlling intracellular and membrane structures, as well as to genes whose deficiency is compensated for by genetic redundancy. Comparable approaches, using efficient phenotyping technology [50] and appropriate screening procedures, could be used for identification of genes involved in specific functions, such as homeostasis and response to stress.

We have carried out an analysis of toxic protein domains, pointing out the importance of binding domains and of protein-protein interactions correlated to regulation of cell growth and cell division. This provides a large body of data for targeting more specific studies on the modular construction of proteins and the role of interaction domains in multicomponent assembly of physiological complexes. Finally, in some cases, the deleterious effects in our system of inserts that encode very short ORFs may suggest that overexpression of some transcripts is also toxic for cell growth.

## Materials and methods

### Strains and media

Total yeast DNA from strain FY1679 (*Mata/α, ura3-52/ura3-52, trp1-Δ63/+, leu2-Δ1/+, his3-Δ200/+*) [51] was a generous gift of A. Harington. Strains FYBL2-5D (*Matα, ura3-Δ851, trp1-Δ63, leu2-Δ1*) [52] and FYAT-01 (*Matα, ura3-Δ851, trp1-Δ63, leu2-Δ1, his3-Δ200, ade2-661*) (A. Thierry, unpublished work) were used for transformations and growth defect screening. All strains are isogenic derivatives of S288C.

The yeast genomic library was constructed using *Escherichia coli *DH10B cells (Electromax DH10B, Gibco-BRL).

Yeast cells transformed by pCMha190 recombinants were grown at 30°C on glucose synthetic complete medium lacking uracil (SC - URA) always supplemented with 10 μg/ml doxycycline (Sigma) (uninduced conditions). Phenotypic tests were done on solid medium (12 cm × 12 cm plates) containing 70 ml of SC - URA + 10 μg/ml doxycycline (uninduced conditions) or SC - URA without doxycycline (overexpression conditions). Yeast cells transformed by pCMha191 recombinants were grown at 30°C on SC - tryptophan medium, with or without addition of doxycycline. Plasmid loss was carried out on SC plates containing uracil (50 mg/l) and 0.1% of 5-fluoroorotic acid (5-FOA).

### Vector construction and cloning

Plasmids pCMha184, pCMha189, and pCMha190 were derived from the centromeric (pCM184, pCM189) or episomal (pCM190) overexpression vectors, containing a tetracycline-regulatable promoter system and *URA3 *(pCM189, pCM190) or *TRP1 *(pCM184) as selection markers [31]. In the original vectors, a 33 bp *Bam*HI-*Not*I fragment was replaced by a synthetic linker with ends compatible with these sites and introducing an ATG codon followed by an in-frame HA-tag, a *Bam*HI cloning site, and stop codons in the three frames (Figure 1a). The episomal pCMha191 vector was derived from pCMha184 (*TRP1 *selection marker) by replacement of the centromere and replication origin with a 2 μm plasmid replication origin. This was PCR-amplified from pCMha190 using primers M1 and M2 (see Additional data file 8) using *Pfu *polymerase (Stratagene), and ligated to the 5,953 bp *Eco*RI-*Bgl*II fragment of pCMha184.

The overexpression system was checked by cloning two short genes, *MCM1 *and *AUAI *(861 and 285 bp respectively), which are toxic when overexpressed under the control of a *GAL1 *promoter [29]. Both genes were PCR-amplified from yeast genomic DNA (see primers in Additional data file 8), cloned into vectors pCMha189 and pCMha190, and transformed into yeast strain FYAT-01. Only gene *MCM1*, cloned in the high-copy pCMha190 vector, had a clear and constant toxic effect on yeast growth when overexpressed. We thus decided to build the library into pCMha190 and to choose the *MCM1 *gene as a control for toxic phenotype in overexpression conditions.

Thirteen complete genes corresponding to 13 selected toxic inserts (see Results) and the *MCM1 *control gene were cloned into the *Bam*HI digested plasmid pCMha191 (*TRP1 *marker)*. *Genes were PCR-amplified from genomic DNA (see primers in Additional data file 8). For each gene, two independent plasmids were transformed into yeast strain FYBL2-5D. In parallel, the same strain was transformed with the plasmids bearing the corresponding toxic inserts.

Two toxic inserts, 156C1 and 57B6, which are antiparallel fragments of *YGL039w *and *YAL062w/GDH3 *ORFs, were modified by PCR synthesis (see Additional data file 9), then recloned *in vivo *into pCMha190 using homologous recombination [53] in yeast strain FYBL2-5D. Constructions were verified by sequencing. In parallel, original plasmids extracted from transformed strain FYAT-01 were retransformed into strain FYBL2-5D. Phenotypes in uninduced and overexpression conditions were observed in seven independent transformants in each case.

### Construction of a random yeast genomic library into pCMha190

The adaptor-based strategy [7,54] was used to prevent self-ligation of the vector and ligation of multiple inserts.

Sonicated total yeast DNA fragments from FY1679 ranging in size from 200 to 1,200 bp were treated with mung-bean nuclease, T4 DNA polymerase and Klenow enzyme following the manufacturers' protocols. Blunt ends of DNA fragments were ligated to the following adaptor:

5'-pATCCCGGACGAAGGCC-3'

3'-GGCCTGCTTCCGG-5'.

Excess of unligated adaptors and small adaptor-DNA fragments were eliminated by two consecutive purifications using Chroma spin+TE-400 columns (Clontech). Vector predigested with *Bam*HI and filled in with dGTP by the Vent (exo-) polymerase (New England Biolabs) was ligated to the purified adaptor-DNA inserts (800 ng = ~0.16 pmol vector, 800 ng = ~1.7 pmol inserts, in a 40 μl final volume per ligation). The ligation result is drawn in Figure 1b.

Electroporations of 40 μl of *E. coli *DH10B cells were performed with 1.8 μl of ligation mix and plated onto 2YT medium (16.1 g/l Bacto tryptone, 10.1 g/l Bacto yeast extract, 5 g/l NaCl, 15 g/l Bacto agar) containing 100 μg/ml ampicillin (four 12 × 12 cm plates per transformation) giving 25,000 to 45,000 clones per transformation.

A total of 51 independent transformations were made. This corresponds to 1,888,000 clones. We tested 150 clones for the presence of an insert and observed that more than 85% contained one (average size 700 bp, minimum 220 bp, maximum 1,620 bp). Colonies from each transformation were pooled and distinct Qiagen Tip 500 DNA preparations were made and stored separately for yeast transformation. Final concentration of DNA was 300 to 1,300 ng/μl. The detailed protocol of library construction is available on request.

Another library had previously been constructed with the same vector ligated to a distinct DNA-adaptor preparation and was partially used, giving rise to 160,000 primary clones. Characteristics of the transformants were the same as described above. Eight pools of plasmid DNA were prepared from this first library.

### Yeast transformations

We carried out a total of 28 independent transformations of yeast by the LiAc method [55]: five with the yeast strain FYAT-01 using five distinct plasmid DNA preparations from the first library and 23 with the strain FYBL2-5D using 23 distinct plasmid DNA preparations from the main library. Aliquots of each transformation were spread onto 24 × 24 cm plates (Q-Pix Trays, Genetix) containing SC - URA + doxycycline, to obtain 1,000 to 3,000 yeast transformants per plate.

### Screening and storage of toxic clones

Transformed yeast clones were transferred into fresh liquid SC - URA + doxycycline medium in 96-well microplates by manual picking (30,015 clones) or with the Q-Pix robot (54,071 clones) for overnight growth. Non-toxic and toxic control clones (transformed by empty pCMha190 vector and by vector bearing *MCM1*, respectively) were also inoculated into each microplate. Cultures were grown overnight at 30°C and stored at 4°C before dilutions for phenotypic examination. Screening of the toxic phenotypes after overexpression was done in a two-round selection, using the 'drop test', which allowed us to see even slightly impaired growth effects. Ten-fold serial dilutions in water were made from each 96-well culture microplate with a Beckman Biomek 2000 robot, then manually replicated with the 96-pin Beckman replicator onto SC - URA + doxycycline and SC - URA plates in parallel (Figure 1c). Clones showing impaired growth in overexpression conditions were streaked onto SC - URA + doxycycline medium for colony isolation, then transferred (one subclone per streak) into a new 96-well microplate and grown for 22 h at 30°C. This plate served as a mother plate for four culture microplates which were grown overnight at 30°C (one plate for the second-round screening, another plate for PCR amplification on colonies for sizing and sequencing the inserts and two plates for storage at -80°C). For the second round of screening, cultures were diluted (1/100 to 1/10,000 dilution) and tested on SC - URA + doxycycline and SC - URA plates in parallel. Phenotypes in the presence and absence of doxycycline (uninduced and overexpression conditions respectively) were scored as described in Results and Figure 2. Between the two rounds of screening, most of the clones conserved a comparable phenotype. For those displaying an important difference, a new subclone was tested again, and the transformant was rejected if the phenotype revealed was inconsistent.

The dependence of the phenotype on the presence of the plasmid was demonstrated using two methods: for 150 tested clones, wild-type phenotypes were recovered after plasmid loss using 5-FOA resistance selection; for 35 other clones, plasmids were extracted from transformed strain FYAT-01 and retransformed into strain FYBL2-5D, in which the toxic phenotypes were confirmed.

### Identification of the toxic inserts at the nucleotide and peptide levels

Inserts of the selected clones were PCR-amplified directly from cultures using primers SEQ4 and SEQ8 (Figure 1b). The length of each insert was determined by gel electrophoresis and the 5' junction was sequenced using primer SEQ1. Identification of each insert in our internal database (see below) was carried out using the DOGEL program [56], adapted by Nicolas Joly (Institut Pasteur) to our purpose. This program gives the start position of the insert on chromosomes, the corresponding genetic object and the start position in the ORF relative to the natural ATG (see Additional data files 1 and 2). We first verified the sequence at the junction with the adaptor-insert. Correct in-frame ligation between vector and adaptor-1 was observed for 632 clones (88.5% of total). For the remaining 82 clones, base substitutions, and short (one to three nucleotides) deletions within the adaptor-1 were observed (nine and 18 cases respectively). A total of 46 cases of a single G addition at the junction vector-adaptor-1, and 15 partial vector sequence duplicates were found (see Additional data files 1 and 2). As the incorrect ligations introduced no stop codon between the initiation codon of the vector and the first codon of the insert, these clones were conserved for further analysis. In these cases, the start position of the insert relative to the chromosome and to the ORF coordinates was corrected manually.

For analysis of in-frame ORF fragments, sequences of peptides encoded by toxic inserts were extracted from the complete sequences of *S. cerevisiae *proteins, taking the first amino acid corresponding to the junction with the adaptor as the starting point and the end of the insert or the last codon of the ORF as the end point.

Fragments of mtDNA, 2 μm plasmid, and DNA coding for Y'-ORFs, Tys, long terminal repeats (LTRs) and RNA were examined manually for their position relative to the coding sequences.

Sequences of inserts other than in-frame ORF fragments were systematically translated into amino-acid sequences from the junction with the adaptor up to the first stop codon encountered in the insert. Sequences coding for more than 24 amino acids were internally compared using BLASTP, then compared to the *S. cerevisiae *annotated ORFs and to the 308,738 sequences of our internal database (see below).

### Databases

Genetic entities corresponding to the toxic inserts were identified by comparison with the DNA sequences of the 16 chromosomes (available in the Comprehensive Yeast Genome Database (CYGD) at MIPS [38]); with our own interpretation table containing the coordinates of 6,256 coding sequences (CDS or ORFs), which comprises the new genes found by Blandin *et al. *[57]; with the 2 μm plasmid DNA sequence [58]; and with the yeast mitochondrial sequence [59]. The set of 6,256 ORFs of *S. cerevisiae *was filtered to eliminate all spurious ORFs or unlikely real genes, as well as Ty, Y' and mitochondrial ORFs, yielding a final list of 5,803 ORFs [60]. For all comparisons of the set of 454 toxic ORFs with the set of ORFs of the entire genome, we used these 5,803 ORFs. GPROTEOME3 is an updated version of the GPROTEOME sequence library [61] containing 308,738 predicted protein sequences from 60 organisms (F. Tekaia, personal communication).

### Analysis of the toxic inserts and of their cognate genes

Comparisons among the peptides encoded by in-frame ORF fragments were done using BLASTP [62]. Alignments corresponding to E-values equal to or lower than 10^-3 ^were examined individually before validation.

Conserved domains or patterns of COGs [32] were identified using the NCBI Conserved Domain Search service (CD-Search [63,64]). The NCBI Conserved Domain Database (cdd.v1.62) [65] contained domains derived from Smart [66] and Pfam [67] collections, plus contributions from NCBI such as COGs, leading to 11,088 position-specific score matrices (PSSMs). A routine was written for extraction of the CD-Search results obtained for the toxic inserts and the 5,803 proteins of the entire genome. The cut-off E-value was chosen to be equal to or less than 10^-4 ^for most domains, and 10^-3 ^for short domains (60 amino acids or fewer). Domains were considered as present even when represented only partially. In describing genes (Table 4) or toxic in-frame inserts (see Additional data files 1, 3 and 4), only one domain (giving the best hit) was chosen for a given insert, among several possible hits. In contrast, to compare the frequency of a given domain among all toxic inserts versus its frequency among the 5,803 proteins of *S. cerevisiae *(Table 2), all occurrences were taken into account, giving a total of 843 occurrences among the 493 toxic inserts, and a total of 15,925 occurrences among the 5,803 proteins.

Searches for transmembrane spans (TMS) were done using TopPredII [68] implemented by Deveaud and Schuerer (Institut Pasteur), predicting both certain and putative TMS. The isoelectric points (IEPs) of proteins or peptides were calculated using iep algorithm from the European Molecular Biology Open Software Suite (EMBOSS) [69].

Descriptions of selected genes and their products were retrieved from the Yeast Proteome Database [70] (release of March 2002; this database is no longer freely available), and from MIPS [38]. Functional classes, cellular localizations and a list of essential genes were retrieved from MIPS [38]; gene classes (conserved/asco-specific/orphan) are from Génolevures [37]. Paralogous gene families of *S. cerevisiae *[57] are accessible at Génolevures [37] through gene or ORF name.

We searched for the participation of the selected ORFs in protein-protein interactions (genetic and physical) and in protein complexes using three different sources: YPD [70] files for individual proteins; protein complexes defined by Gavin *et al. *[10]; data compilations concerning protein-protein interactions and complexes, extracted from SGD [1], MIPS [38] and unpublished two-hybrid experiments (M. Fromont-Racine and C. Saveanu, personal communication).

### How representative is our screening?

We consider that our library contains DNA fragments randomly distributed throughout the genome. Out of 84,086 clones tested, 11% (9,530) contain a DNA fragment cloned in-frame with the frame of the natural ORF (~68% of the genome corresponds to coding regions, and only one frame out of six corresponds to the natural frame), the others containing noncoding, out-of-frame or antisense DNA fragments. If we use the simplifying assumption that all genes are equally represented among the 9,530 clones (not taking into account the size diversity of genes), each of the 5,803 ORFs will be represented 1.64 times (9,530/5,803). The probability *P*_x _of encountering any gene *x *times is described by a Poisson distribution:



where *m*, the mean of the distribution, is 1.64. This is used to estimate the fraction of genes not encountered: for *x *= 0 and probability *p *= 0.19, the number of non-encountered genes = 1,126. Thus, by screening a total of 84,086 clones, we have encountered a maximum of 4,677 ORFs (5,803 - 1,126).

## Additional data files

The following additional data are available with the online version of this article. Additional data file 1 contains lists and coordinates of the 493 in-frame fragments of annotated ORFs giving toxic phenotypes when overexpressed, and short description of their cognate genes. Additional data file 2 contains a list and description of the 221 DNA toxic inserts other than in-frame ORF fragments. Additional data file 3 gives a description of the peptides encoded by the 493 toxic ORF fragments, and of the cognate proteins. Additional data file 4 gives the content of the 57 groups of peptide inserts sharing similarities. Additional data file 5 gives a list and description of protein domains found only once among the toxic inserts. Additional data file 6 lists the genes selected in this work whose products are members of complexes [10]. Additional data file 7 lists genes selected in this work whose products are known as interacting with each other. Additional data file 8 contains the sequences of the oligonucleotides used in this work. Additional data file 9 contains a figure showing the phenotypes induced by overexpression of antiparallel ORF fragments before and after introduction of a stop codon upstream of the artificial ORFs.

## Supplementary Material

Additional data file 1 Lists and coordinates of the 493 in-frame fragments of annotated ORFs giving toxic phenotypes when overexpressed, and short description of their cognate genes Click here for additional data file

Additional data file 2 A list and description of the 221 DNA toxic inserts other than in-frame ORF fragments Click here for additional data file

Additional data file 3 A description of the peptides encoded by the 493 toxic ORF fragments, and of the cognate proteins Click here for additional data file

Additional data file 4 The content of the 57 groups of peptide inserts sharing similarities Click here for additional data file

Additional data file 5 A list and description of protein domains found only once among the toxic inserts Click here for additional data file

Additional data file 6 The genes selected in this work whose products are members of complexes Click here for additional data file

Additional data file 7 Genes selected in this work whose products are known as interacting with each other Click here for additional data file

Additional data file 8 The sequences of the oligonucleotides used in this work Click here for additional data file

Additional data file 9 A figure showing the phenotypes induced by overexpression of antiparallel ORF fragments before and after introduction of a stop codon upstream of the artificial ORFs Click here for additional data file

## Figures and Tables

**Table 1:** Distribution of the toxic inserts between the different genetic objects

Genetic objects represented	Number of toxic inserts	Percentage of total	Mean size ± SD (nucleotides) (minimum-maximum)	Phenotypes				Inserts encoding artificial peptides
					
				3/0, 3/1	3/2	2/0, 2/1	1/0	
In-frame ORF fragments	493	68.7	743 ± 311 (220-2,120)	375	87	23	8	_
Antiparallel ORF fragments	68	9.6	532 ± 247 (140-1,220)	37	11	12	8	53
Out-of-frame ORF fragments	53	7.5	733 ± 306 (170-1,620)	12	11	22	8	12
Intergenic regions	41	6.0	625 ± 358 (170-1,820)	13	4	16	8	27
LTRs	2	0.3	595 (320-1,120)	1	0	0	1	1
Ty elements	15 (10)	2.1	633 ± 265 (320-870)	7	4	2	2	_
Y' elements	9 (3)	1.2	678 ± 370 (320-1,320)	9	0	0	0	6
RNA genes	4	0.5	662 ± 246 (470-1,020)	3	0	1	0	3
2 μm plasmid	17 (10)	2.4	564 ± 288 (170-1,220)	13	3	1	0	5
Mitochondrial DNA	12	1.7	483 ± 201 (200-920)	9	3	0	0	10
Total	714	100	703 ± 313 (140-2,120)	479	123	77	35	117

The first column indicates nature of sequence in toxic inserts. Second and third columns contain, respectively, actual number of inserts of each type and corresponding percentages. For Tys, Y' and 2 μm plasmid, numbers in brackets represent numbers of in-frame fragments of natural ORFs. The fourth column shows the mean size of insert in nucleotides ± standard deviation (SD) with minimum and maximum sizes in brackets. Scoring of each type of phenotype is shown in the next four columns. The last column shows the number of inserts in which artificial ORFs of more than 24 codons were detected.

**Table 2:** Conserved domains found more than once among the toxic in-frame ORF fragments

Domain reference	Domain name	*S. cerevisiae*	Toxic inserts	Mean	95% confidence interval	Result	Domain description
**Transport-specific domains**							
COG0471	CitT	4	4	0.21	0.17-1.25	+	Di-and tricarboxylate transporter
pfam03169	OPT	3	3	0.16	0.11-1.17	+	Oligopeptide transporter protein
COG1953	FUI1	9	3	0.48	0.44-1.56	+	Nucleotide transporter
pfam00324	aa_permeases	22	7	1.16	1.04-2.22	+	Amino acid permease
pfam00153	mito_carr	97	24	5.13	5.07-6.45	+	Mitochondrial carrier protein
COG0531	PotE	26	5	1.38	1.28-2.48	+	Amino acid transporter
COG0474	MgtA	23	4	1.22	1.12-2.30	+	Cation transport ATPase
cd00267	ABC_ATPase	58	6	3.07	2.93-4.22	+	ABC transporter nucleotide-binding domain
pfam00664	ABC_membrane	14	2	0.74	0.68-1.82	+	ABC transporter transmembrane region
COG0842	COG0842	6	3	0.32	0.29-1.38	+	ABC-type multidrug transport system, permease component
COG1131	CcmA	54	4	2.86	2.74-4.01	NS	ABC-type multidrug transport system, ATPase component
pfam00083	Sugar_tr	58	5	3.07	2.94-4.23	+	Sugar (and other) transporter
							
**RNA-and DNA-binding domains**							
pfam00076	rrm	72	11	3.81	3.62-4.95	+	RNA recognition motif (transcription)
COG5099	(PUF)	9	5	0.48	0.44-1.56	+	Pumilio family RNA-binding repeat (translational repression)
smart00322	KH	11	4	0.58	0.54-1.66	+	K homology: RNA-binding domain (transcription, RNA metabolism)
smart00356	ZnF_C3H1	5	4	0.26	0.21-1.30	+	Zinc finger, C3H1 type (transcription)
COG5048	C2H2-type Zn_finger	15	4	0.79	0.74-1.89	+	Zn-finger (C2H2-type) (transcription)
COG0210	UvrD	4	2	0.21	0.17-1.24	+	DNA and RNA helicases, superfamily I (DNA replication, recombination, repair)
cd00086	Homeodomain	9	2	0.48	0.45-1.57	+	DNA binding domain (eukaryotic development)
pfam00249	myb_DNA-binding	13	2	0.69	0.66-1.80	+	Myb-like DNA-binding domain (transcription)
pfam00170	bZIP	4	2	0.21	0.17-1.25	+	Basic-leucine zipper DNA binding and dimerization domains (transcription)
smart00066	GAL4	48	2	2.54	2.44-3.72	NS	GAL4-like Zn(II)2Cys6 DNA-binding domain (fungal) (transcription)
pfam04082	Fungal_trans	26	2	1.38	1.29-2.48	NS	Fungal specific transcription factor domain.
pfam00270	DEAD	48	3	2.54	2.38-3.63	NS	DEAD/DEAH box helicase (replication, repair, transcription)
cd00079	HELICc	60	2	3.18	3.08-4.34	_	Helicase superfamily, C-ter domain (replication, repair, transcription)
							
**Domains involved in Interactions with peptides, proteins or phospholipids**							
cd00200	WD40	327	29	17.31	16.87-18.54	+	Tandem repeats of about 40 residues interacting with peptides
pfam01602	Adaptin_N	9	2	0.48	0.43-1.54	+	N-ter region of adaptor proteins (clathrin-coated pits and vesicles)
pfam00786	PBD	4	2	0.21	0.20-1.27	+	P21-Rho-binding domain (or CRIB)
pfam00169	PH	11	3	0.58	0.55-1.67	+	PH: pleckstrin homology. binds phosphoinositides or other ligands (signalling)
COG5271	MDN1	16	3	0.85	0.78-1.93	+	AAA : ATPase with von Willebrand factor type A domain (multiprot. complexes)
smart00268	ACTIN	14	2	0.74	0.67-1.82	+	ACTIN, cytoskeleton/motor protein
COG5022	Myosin heavy chain	7	5	0.37	0.33-1.43	+	ATPase, molecular motor
COG5043	MRS6	4	2	0.21	0.17-1.24	+	Vacuolar protein sorting-associated protein
KOG0446*	Dynamin	3	3	0.16	0.13-1.20	+	GTPase that mediates vesicle trafficking
							
**Metabolism-related domains**							
pfam03901	PMP	5	2	0.21	0.21-1.29	+	Mannosyltransferase
COG1928	PMT1	7	4	0.37	0.30-1.40	+	Mannosyltransferase
pfam00561	Abhydrolase	18	3	0.95	0.88-2.05	+	Abhydrolase, alpha/beta hydrolase fold (catalytic domain)
pfam00107	ADH_zinc_N	21	2	1.11	1.01-2.19	NS	Zinc-binding dehydrogenase
pfam00501	AMP-binding	11	2	0.58	0.51-1.64	+	AMP-binding synthetase
							
**Other domains**							
pfam00674	DUP	35	3	1.85	1.81-3.03	NS	DUP family (proteins of unknown functions)
COG5384	Mpp10	1	2	0.05	0.03-1.07	+	M phase phosphoprotein 10 (U3 small nucleolar ribonucleoprotein component)
COG5032	TEL1	8	4	0.42	0.34-1.44	+	PI kinase and protein kinases of the PI kinase family
COG1025	Ptr	5	2	0.26	0.22-1.31	+	Zn-dependent peptidases (secreted/periplasmic, insulinase-like)
pfam02902	Peptidase_C48	2	2	0.11	0.08-1.13	+	Ulp1 protease family, C-terminal catalytic domain
pfam00004	AAA	43	3	2.28	2.15-3.39	NS	AAA, ATPase family associated with various cellular activities (AAA)
smart00220	S_TKc	125	4	6.52	6.31-7.72	-	Serine/threonine protein kinases, catalytic domain

Peptide sequences of toxic natural ORF fragments were searched for domains (see text), and the frequency of domains found more than once was compared to the frequency in the whole proteome. References and names of domains are in the first two columns; occurrences in the whole genome (*S. cerevisiae*) and in the toxic inserts are in the third and fourth columns, respectively. The next three columns show the statistical analysis performed as follows: 1,000 random selections of 843 domains (total number of occurrences in the toxic inserts) were made from the set of 15,925 domains identified in *S. cerevisiae *(see Materials and methods); mean (column 5) represents the mean number of occurrences of each domain among the toxic inserts; the 95% confidence interval (column 6) was calculated using the SD of the 1,000 random drawings; column 7 shows the result of this analysis for each domain: NS, not significant; +, domain over-represented in toxic inserts; -, domain under-represented in toxic inserts. The last column gives a brief description of domains from NCBI Conserved Domain Database [65]. *KOG0446 was found using cdd.v1.63 of NCBI CD-Search [64].

**Table 3:** Distribution of selected genes versus all *S. cerevisiae *genes

	All *S. cerevisiae *genes	Percentage of total	Selected toxic genes	Percentage of total
**Functional classes (MIPS data)**				
**Cell cycle_DNA processing**	670	**11.5**	75	16.5*
**Cell fate**	486	**8.4**	66	14.5*
Cell rescue, defense and virulence	288	5.0	23	5.1
Cellular communication/signal transduction mechanism	59	1.0	6	1.3
**Cellular transport and transport mechanisms**	525	**9.0**	67	14.8*
Classification not yet clear-cut	112	1.9	6	1.3
Control of cellular organization	207	3.6	22	4.8
Energy	244	4.2	12	2.6
Metabolism	1,061	18.3	88	19.4
Protein fate (folding, modification, destination)	593	10.2	47	10.4
**Protein synthesis**	377	**6.5**	17	3.7*
**Regulation of/interaction with cell. Environment**	197	**3.4**	29	**6.4**^†^
**Transcription**	801	**13.8**	88	19.4*
**Transport facilitation**	321	**5.5**	61	13.4*
**Unclassified**	1,706	**29.4**	91	20.0*
				
**Cellular localization (MIPS data)**				
Extracellular	54	1.4	5	1.6
Cell wall	38	1.0	4	1.3
Golgi	103	2.6	8	2.5
Transport vesicles	54	1.4	3	0.9
**Plasma membrane**	171	**4.4**	34	10.7*
**Nucleus**	1,367	**34.8**	130	**40.8**^†^
**Cytoplasm**	2,001	**50.9**	137	42.9*
Peroxisome	42	1.1	3	0.9
Endosome	20	0.5	2	0.6
**Cytoskeleton**	154	**3.9**	22	**6.9**^†^
Vacuole	82	2.1	8	2.5
Endoplasmic reticulum	353	9.0	27	8.5
Mitochondria	562	14.3	37	11.6
				
**Viability (MIPS data)**				
**Essential**	939	**16.2**	96	**21.1**^†^
Essential or not	160	2.8	20	4.4
				
**Phylogeny (Génolevures data)**				
**Conserved**	3,717	**64.1**	336	74.0*
**Ascomycete-specifics**	1674	**28.8**	106	23.3*
**Orphan**	412	**7.1**	10	2.2*

The distribution of genes was examined in respect of four classifications: function, cellular localization of the gene product, viability and phylogeny. Data are from MIPS [38] and Génolevures [37]. Cellular localization was known for 3,928 out of the 5,803 proteins in the entire genome and for 319 proteins out of the 454 that yield toxic inserts. For other comparisons, the set of 454 selected genes was compared to the set of 5,803 genes of *S. cerevisiae*. Note that a given gene may be present in more than one MIPS class. Significant evidence that a given gene class is over-or under-represented among toxic genes as compared to all *S. cerevisiae *genes is emphasized by bold characters. *p < 0.005; ^†^*p *< 0.025.

**Table 4:** Toxicity of fragments versus whole ORF products

ORF/Gene name	Gene description	Phenotype of gene deletion	Conserved domain or TMS in entire protein	Phenotype of gene overexpression	Conserved domain or TMS in insert	Phenotype of insert overexpression
YDL112w/TRM3*	tRNA 2'-O-ribose methyltransferase	Viable	SpoU_methylase	3/3	-	3/1
YML128C/MSC1/ GIN3*^†^	Weak similarity to *Schizosaccharomyces pombe *stress protein	Viable	1 TMS	3/3	-	3/0
YGR149w/_* ^‡§^	Similar to *S. pombe *hypothetical protein	Viable	5 TMS	3/2 to 3/3	3 TMS	3/2
YGL023c/PIB2* ^§^	Phosphatidylinositol 3-phosphate binding	Viable	FYVE	3/1	FYVE	3/0
YPL043w/NOP4^¶^	Nucleolar protein, RNA processing	Lethal	RRM (4 motifs)	3/0	Bias D, E, K	3/0
YOR166c/_ * ^§^	Similarity to hypothetical *S. pombe *protein	Viable	PINc (nucleotide binding)	3/0	PINc	3/0
YJL212c/OPT1^¶¥^	Oligopeptide transporter	Viable	OPT	3/1	2 TMS, OPT	3/1
YNL003c/PET8^¥¤^	Mitochondrial carrier	Viable	mito_carrier	3/2	mito_carrier	3/2
YJL092w/HPR5^¥#^	DNA helicase involved in DNA repair	Viable	UvrD	2/0	UvrD (central)	3/2
YMR190c/SGS1^¥¤^	DNA helicase of DEAD/DEAH family	Viable	DEAD, HELICc, HRDC	3/0	DEAD	3/2
YNL033W/_^§^	Strong similarity to YNL019c	Viable	2 TMS	3/1	1 TMS	3/2
YHR067w/_* ^§^	Weak similarity to *S. pombe *hypothetical protein	Viable	Maoc : Acyldehydratase	3/1	MaoC	3/2
YGL263w/COS12^§¥¤^	Similarity to subtelomeric encoded proteins	Viable	DUP	3/0	DUP	3/1

Systematic nomenclature and gene name, where applicable, are given in the first column. *Singleton: the gene has no paralog in *S. cerevisiae*. ^†^Gene fragment and ^#^entire gene, respectively, were already known as toxic upon overexpression. ^‡^Putative uncharacterized transporter (see [35]). ^§^Gene of unknown classification. ^¶^Two non-overlapping inserts of the ORF were selected. ^¥^One or several paralogs of this gene have also been selected as toxic inserts in this work (see Additional data file 3). ^¤^Gene having a paralog in *S. cerevisiae *already known as toxic upon overexpression. Columns 2 and 3 contain respectively a brief description of the function of the gene product and the phenotype of the disruption mutant (MIPS [38]). The results of a search for conserved domains is shown in columns 4 (in whole protein) and 6 (in inserts). Phenotypes in uninduced and overexpression conditions of the entire gene and of fragments are given in columns 5 and 7 respectively (see Figure 3 for illustrations of the phenotypes).
