# Supplementary material for: Large-scale exploration of growth inhibition caused by overexpression of genomic fragments in Saccharomyces cerevisiae
Source: Genome Biol. 2004 Aug 31;5(9):R72. doi: 10.1186/gb-2004-5-9-r72 (PMC522879; doi:10.1186/gb-2004-5-9-r72)
Supplement: Additional data file 9 — A figure showing the phenotypes induced by overexpression of antiparallel ORF fragments before and after introduction of a stop codon upstream of the artificial ORFs [file gb-2004-5-9-r72-s9.pdf]

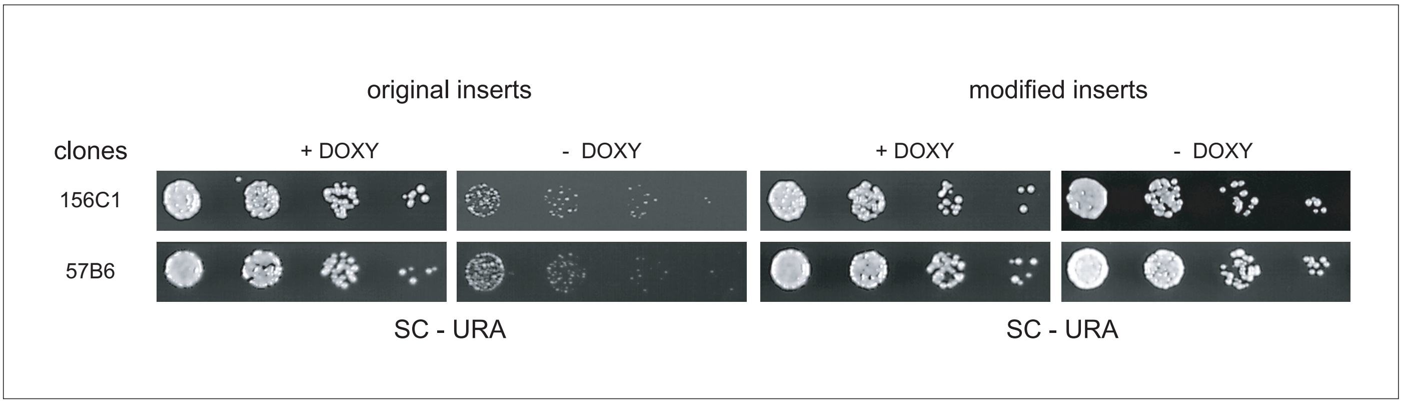

Additional data file 9: Figure S1. Phenoetypes induced by overexpression of antiparallel ORF fragments before and after introduction of a STOP codon

Original inserts 156C1 and 57B6 are antiparallel fragments from ORFs YGL039w and YAL062w respectively. They encode artificial ORFs of 73 and 102 codons respectively. They were modified by PCR using the primers below, and recloned into pCMha190 (see Materials and methods). Left panel shows original toxic phenotypes and right panel shows phenotypes after modification.

156C1_upstream: 5'-CCCAGACTACGCTGGATCCCGGACGAAGGCCtaaatt**GGAATCCTAATAAATTTTTGGTTTTGCG**-3'
156C1_downstream: 5'-GCGGCCCTCCTGCAGGGCCCTAGCGGCCG**CGCGATGTTTCAAAAGCTCATTTACTTGC**-3'
57B6_ upstream: 5'-CCCAGACTACGCTGGATCCCGGACGAAGGCCtagctgcctttggtggcc**CAAACCAAACTGCATCC**-3'
57B6_ downstream: 5'-GCGGCCCTCCTGCAGGGCCCTAGCGGCCG**GGGGTGCATCATCTCTGAGACGGGC**-3'

Upper case plain letters correspond to sequences of vector and adaptor, upper case bold letters correspond to sequences of insert, lower case plain letters are the sequences introducing the stop codons, which correspond to a part of the natural upstream sequences of the cloned fragments.

Additional data file 9: Figure S1. Phenotypes induced by overexpression of antiparallel ORF fragments before and after introduction of a STOP codon upstream the arti
